# The complete chloroplast genome of a critically endangered tree species, *Hopea reticulata* (Dipterocarpaceae)

**DOI:** 10.1080/23802359.2020.1829123

**Published:** 2020-10-21

**Authors:** Xiang Ma, Ying Cai, Liang Tang

**Affiliations:** a Key Laboratory of Tropical Biological Resources of Ministry of Education, School of Life and Pharmaceutical Sciences, Hainan University, Haikou, China; b College of Ecology and Environment, Center for Terrestrial Biodiversity of the South China Sea, Hainan University, Haikou, China; c Center for Terrestrial Biodiversity of the South China Sea, Hainan University, Haikou, China

**Keywords:** *Hopea reticulate*, critical endangered species, chloroplast genome, Dipterocarpaceae

## Abstract

*Hopea reticulata* Tardieu is a critically endangered tree species distributed in limited regions in Thailand, Vietnam and Hainan Island of China. In this study, we reported the complete chloroplast genome sequence of *H*. *reticulata* assembled from pair-end reads generated by Illumina sequencing. The circular chloroplast genome is 151,407 bp in length and comprises a large single copy region of 84,352 bp, a small single copy region of 19,733 bp, and a pair of inverted repeats of 23,661 bp each. The complete genome encodes 129 functional genes, including 83 protein-coding genes, 38 tRNA genes, and 8 rRNA genes. The overall GC content of the chloroplast genome was 37.4%. Phylogenetic analysis showed that *H*. *reticulata* is sister to *H*. *hainanensis* with strong bootstrap support.

*Hopea reticulata* Tardieu is a critically endangered tree species in the family Dipterocarpaceae, which is only found in limited regions in Thailand, Vietnam and Hainan Island of China (Ashton 1998). The wood of *H*. *reticulata* is durable and is useful for building houses and bridges as well as making furniture. In addition, bioactive compounds extracted from barks of this species exhibit potent anticancer activities (Ge et al. 2006). More interestingly, *H*. *reticulata* is the only dominant species in lowland tropical forests of Ganshi Mountain on Hainan Island of China (Hu 1997). Studies have been carried out to explore the community structure and dynamics, as well as species diversity of forests dominated by *H*. *reticulata* in Ganshi Mountain (Mao et al. 2014). However, researches aiming at its molecular phylogeny and population history remain rare. Here, we reported the complete chloroplast genome sequences of *H*. *reticulata* to facilitate the molecular evolutionary studies.

Fresh leaves of *H*. *reticulata* were collected from Ganshi Mountain on Hainan Island of China (N 18°23′26.42″, E 109°39′56.86″). The voucher specimen (HRGS1912) was deposited at the Herbarium of Tropical Plant Research of Hainan University. Total genomic DNA was extracted from silica gel-dried leaves following the modified CTAB method (Doyle and Doyle 1987). A genomic DNA library with an insert size of 400 bp was constructed and then sequenced by an Illumina HiSeq 2500 system at JINTAI Biotech (Guangzhou, China). After adapters and low-quality reads were removed using fastp software (Chen et al. 2018), approximately 2.6 GB paired-end reads were obtained and deposited in the National Center for Biotechnology Information (NCBI) Sequence Read Archive (SRA) under accession number PRJNA662089. Chloroplast genome of *H*. *reticulata* was assembled with GetOrganelle v1.6.2 (Jin et al. 2019) and annotated using the online tool DOGMA (Wyman et al. 2004). The complete chloroplast genome was submitted to GenBank with accession number MT732516.1.

The chloroplast genome of *H*. *reticulata* shows a typical quadripartite structure with a length of 151,407 bp, containing a large single copy (LSC) region of 84,352 bp and a small single copy (SSC) region of 19,733 bp separated by a pair of inverted repeats (IRs) of 23,661 bp each. The overall GC content was 37.4%, while the GC contents of the LSC, SSC, and IR regions were 35.3%, 31.9%, and 43.4%, respectively. The complete chloroplast genome contained 129 genes, including 83 protein-coding genes, 38 tRNA genes, and 8 rRNA genes. 17 genes occurred in the IR region have two copies, including 6 protein-coding genes, 7 tRNA genes, and 4 rRNA genes. As to the 112 unique genes, 14 genes have one intron whereas one gene *ycf*3 has two introns.

To analyze the phylogenetic position of *H*. *reticulata*, complete chloroplast genomes of 14 species belonging to the Dipterocarpaceae and four other related families were retrieved from Genbank. *Arabidopsis thaliana* in the family of Brassicaceae was used as the outgroup. The chloroplast genome alignment was constructed using MAFFT online service (Katoh and Standley 2013). Phylogenetic analysis was performed based on maximum likelihood method implemented in IQ-TREE 1.6.12 (Nguyen et al. 2015) with GTR + F+R4 selected as the best-fit nucleotide substitution model by ModelFinder (Kalyaanamoorthy et al. 2017). The three *Hopea* species form a highly supported monophyletic group, in which *H*. *reticulata* is sister to *H*. *hainanensis* with bootstrap support of 100 (Figure 1). The chloroplast genome of *H. reticulata* provides valuable genetic information for the conservation genetic study on this critically endangered species.

**Figure 1.**
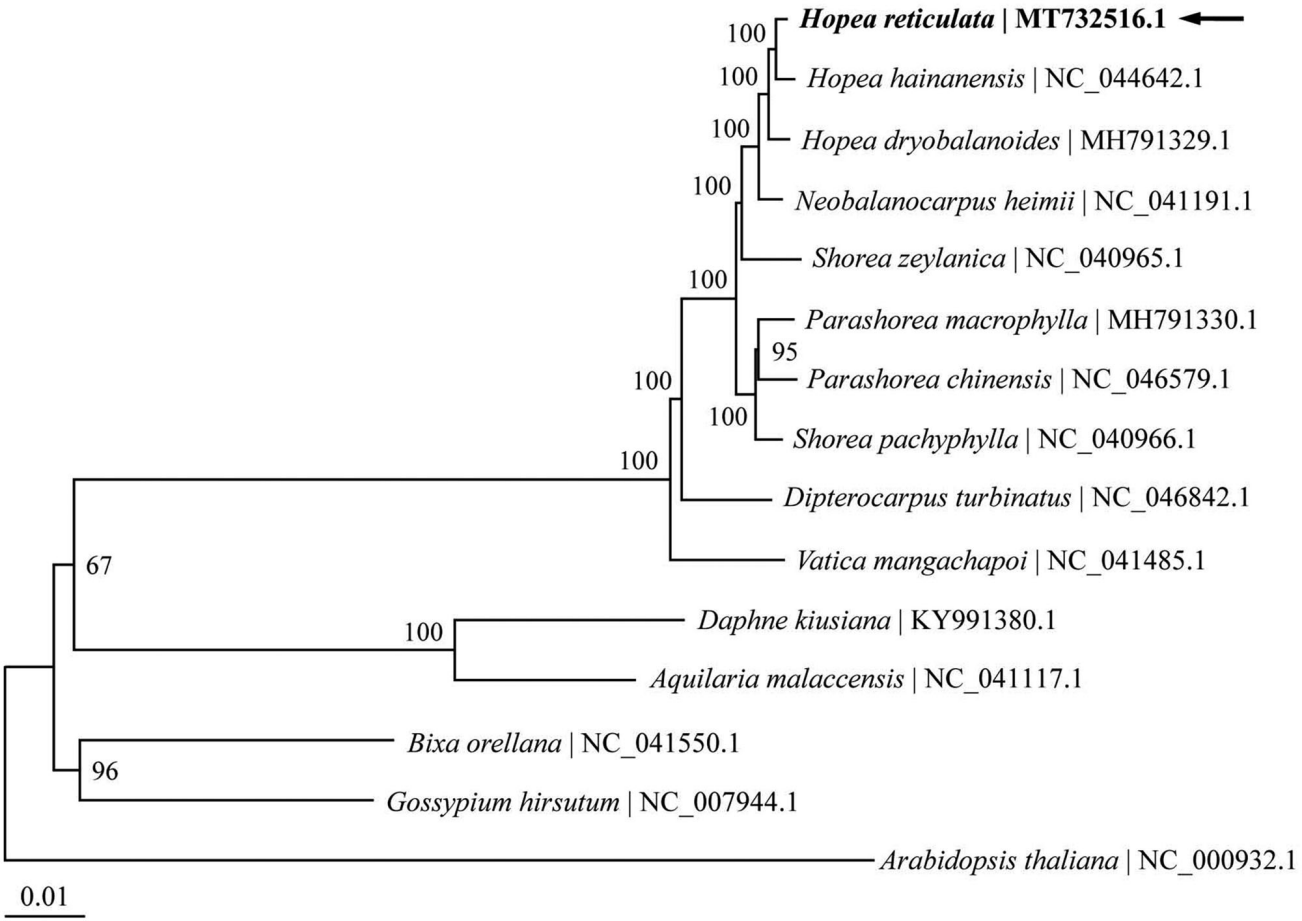
The maximum likelihood tree reconstructed using chloroplast genome sequences of 14 species within the order Malvales plus Arabidopsis thaliana as outgroup. The chloroplast genome of *Hopea reticulata* (MT732516.1) sequenced in this study was highlighted with bold font and an arrow. Numbers above branches are bootstrap support values based on 1000 replicates.

## Data Availability

The data that support the findings of this study are openly available in NCBI at https://www.ncbi.nlm.nih.gov/, reference number [MT732516.1], or available from the corresponding author.
